# Elevated preoperative CEA is associated with subclinical nodal involvement and worse survival in stage I non-small cell lung cancer: a systematic review and meta-analysis

**DOI:** 10.1186/s13019-020-01353-2

**Published:** 2020-10-15

**Authors:** Awrad Nasralla, Jeremy Lee, Jerry Dang, Simon Turner

**Affiliations:** 1 Division of General Surgery, Faculty of Medicine and Dentistry, University of Alberta, Edmonton, Canada; 2 Faculty of Medicine and Dentistry, University of Alberta, Edmonton, Canada; 3 Division of Thoracic Surgery, Faculty of Medicine and Dentistry, University of Alberta, Edmonton, Canada

**Keywords:** Non-small cell lung cancer (NSCLC), carcinoembryonic antigen (CEA), lymph nodes

## Abstract

**Background:**

The standard for clinical staging of lung cancer is the use of CT and PET scans, however, these may underestimate the burden of the disease. The use of serum tumor markers might aid in the detection of subclinical advanced disease. The aim of this study is to review the predictive value of tumor markers in patients with clinical stage I NSCLC.

**Methods:**

A comprehensive search was performed using the Medline, EMBASE, Scopus data bases. Abstracts included based on the following inclusion criteria: 1) adult ≥18 years old, 2) clinical stage I NSCLC, 3) Tumor markers (CEA, SCC, CYFRA 21-1), 4) further imaging or procedure, 5) > 5 patients, 6) articles in English language. The primary outcome of interest was utility of tumour markers for predicting nodal involvement and oncologic outcomes in patients with clinical stage I NSCLC. Secondary outcomes included sub-type of lung cancer, procedure performed, and follow-up duration.

**Results:**

Two hundred seventy articles were screened, 86 studies received full-text assessment for eligibility. Of those, 12 studies were included. Total of 4666 patients were involved. All studies had used CEA, while less than 50% used CYFRA 21-1 or SCC. The most common tumor sub-type was adenocarcinoma, and the most frequently performed procedure was lobectomy. Meta-analysis revealed that higher CEA level is associated with higher rates of lymph node involvement and higher mortality.

**Conclusion:**

There is significant correlation between the CEA level and both nodal involvement and survival. Higher serum CEA is associated with advanced stage, and poor prognosis. Measuring preoperative CEA in patient with early stage NSCLC might help to identify patients with more advanced disease which is not detected by CT scans, and potentially identify candidates for invasive mediastinal lymph node staging, helping to select the most effective therapy for patients with potentially subclinical nodal disease. Further prospective studies are needed to standardize the use of CEA as an adjunct for NSCLC staging.

## Introduction

Lung cancer is the leading cause of cancer related death. Optimal treatment for non-small cell lung cancer (NSCLC) is dependent on accurate clinical staging to determine the extent of disease [1–3]. Patients with lymph node involvement have worse prognosis and may be candidates for neoadjuvant treatment prior to surgical resection. The standard for clinical staging of NSCLC is the use of computed tomography (CT) and positron emission tomography (PET) scans. However, these can underestimate the burden of the disease [4–7]. The median sensitivity and specificity of PET-CT for detection of mediastinal nodal disease is 80 and 88%, respectively [8]. False negatives on imaging studies result in understaging patients who might have benefited from invasive mediastinal staging or neoadjuvant therapy [7, 9, 10]. This had led researchers to investigate the use of biomarkers to increase the sensitivity of clinical staging and allow proper treatment selection. These markers include carcinoembryonic antigen (CEA), squamous cell carcinoma antigen (SCC), and cytokeratin fragment antigen (CYFRA 21-1) [4, 11–13]. To our knowledge this is the first systematic review about the use of tumor markers in patients with clinical stage I NSCLC for predicting lymphatic spread.

## Methods

A comprehensive search was performed for articles published on non-small cell lung cancer and tumor markers using the Medline, EMBASE, Scopus data bases. Search terms included “non-small cell lung cancer” or NSCLC or lung adenocarcinoma, AND carcinoembryonic antigen or squamous cell carcinoma antigen or cytokeratin fragment antigen, AND stage I or stage IA or early stage”. Literature was limited to human studies in the English language. Abstracts and titles were screened for inclusion by two reviewers (AN and JL). Non-relevant articles based on their abstract were not included for full-text evaluation. Abstracts were then further screened based on the following inclusion criteria: 1) adult patient ≥18 years old, 2) primary non-small cell lung cancer (stage I or stage IA or early stage), 3) Tumor markers (Carcinoembryonic antigen (CEA), cytokeratin fragment antigen (CYFRA 21-1) and squamous cell carcinoma antigen (SCC)), 4) any further imaging such as positron emission tomography (PET) scan or procedure such as mediastinoscopy or endobronchial ultrasound (EBUS) or further treatment, 5) studies including > 5 patients, 6) articles in English language. Exclusion criteria included non-English studies, abstracts only, and duplicates.

The primary outcome of interest was utility of tumour markers for predicting pathological tumor invasiveness in patients with clinical stage I NSCLC. Secondary outcomes included subtype of lung cancer, follow-up duration, procedure performed, smoking status, and region of publication. Meta-analysis was performed to determine the following: death within 5 years, and lymph node involvement. This study was conducted and the results are presented according to PRISMA (Preferred Reporting Items for Systematic Reviews and Meta-Analyses) guidelines.

In addition, two independent reviewers assessed the risk of bias using the Newcastle–Ottawa Scale (NOS) for evaluating the quality of the included studies. We rated the quality of the studies (good, fair and poor) according to the guidelines of the NOS. A “good” quality score required 3 or 4 points in selection, 1 or 2 points in comparability, and 2 or 3 points in outcomes. A “fair” quality score required 2 points in selection, 1 or 2 points in comparability, and 2 or 3 points in outcomes. A “poor” quality score reflected 0 or 1 point in selection, or 0 points in comparability, or 0 or 1 point in outcomes (Table 1).

**Table 1:** Risk of bias assessment using NOS

No.	Name of the study	Journal	Quality Score
1	Identifying Patients at Risk of Early Postoperative Recurrence of Lung Cancer: A New Use of the Old CEA Test	Ann Thorac Surg	Good
2	Predictive factors for node metastasis in patients with clinical stage I non-small cell lung cancer	Annals of Thoracic Surgery	Poor
3	Risk Factors for Predicting Occult Lymph Node Metastasis in Patients with Clinical Stage I Non-small Cell Lung Cancer Staged by Integrated Fluorodeoxyglucose Positron Emission Tomography/Computed Tomography	World Journal of Surgery	Good
4	Optimal Predictive Value of Preoperative Serum Carcinoembryonic Antigen for Surgical Outcomes in Stage I Non-Small Cell Lung Cancer: Differences According to Histology and Smoking Status	Journal of Surgical Oncology	Fair
5	Clinical significance of preoperative carcinoembryonic antigen level for clinical stage I non-small cell lung cancer: can preoperative carcinoembryonic antigen level predict pathological stage?	Interactive CardioVascular and Thoracic Surgery	Good
6	Predictive Risk Factors for Mediastinal Lymph Node Metastasis in Clinical Stage IA Non–Small-Cell Lung Cancer Patients	Journal of Thoracic Oncology: Official Publication of the International Association for the Study of Lung Cancer	Fair
7	Sialyl Lewis X as a predictor of skip N2 metastasis in clinical stage IA non-small cell lung cancer	World Journal of Surgical Oncology	Good
8	Clinical significance of preoperative carcinoembryonic antigen level in patients with clinical stage IA non-small cell lung cancer	J Thorac Dis	Good
9	Prognostic impact of Cyfra21–1 and other serum markers in completely resected non-small cell lung cancer	Lung Cancer	Good
10	Significant correlation between urinary N1, N12-diacetylspermine and tumor invasiveness in patients with clinical stage IA non-small cell lung cancer	BMC Cancer	Poor
11	Prediction of lymph node status in clinical stage IA squamous cell carcinoma of the lung	European Journal of Cardio-Thoracic Surgery	Good
12	Predictive Factors for Lymph Node Metastasis in Clinical Stage IA Lung Adenocarcinoma	Annals of Thoracic Surgery	Poor

## Results

### Study selection

**Fig. 1:**
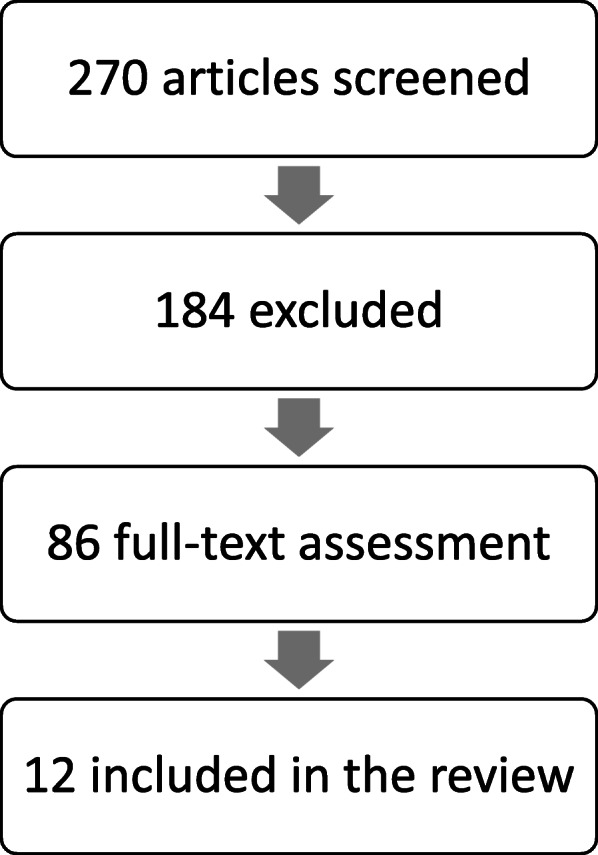
PRISMA diagram with search results for systematic review

Preliminary literature search yielded 270 articles after duplicates were removed. All these 270 studies were screened. Eighty-six studies received full-text assessment for eligibility. Of those, 12 studies were included in the final systematic review (Fig. 1).

### Basic demographics

Twelve studies with 4666 patients were included for systematic review. The majority of these studies were retrospective (10/12) and most were conducted in Japan (8/12). The mean age of the subjects was 65.3 ± 3.2 years, 2602 were males, and 2064 were females (Table 2). The mean follow-up period was 48.86 months. Nine studies, involving 3842 patients reported smoking status, in which 2003 were smokers (52.1%).

**Table 2:** Patients demographics

Number of patients	4666
Male	2602
Female	2064
Age (mean)	65.3 ± 3.2 years
Smoking status	
Smoker	2003
Non-smoker	1839
Not specified	824
Histological types	
Adenocarcinoma	3622
Squamous cell cancer	697
Large cell cancer	54
Adenosquamous carcinoma	17
Carcinoid tumor	12
Others	264
Follow up (mean)	48.86 months
Country	
Japan	8
China	1
Korea	1
Germany	1
Italy	1

### Reporting of tumor markers and tumor characteristics

**Fig. 2:**
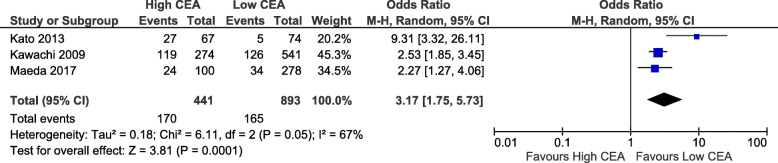
Correlation between CEA level and 5-year mortality

**Fig. 3:**
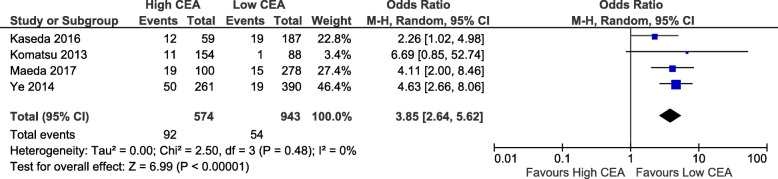
Correlation between CEA level and lymph nodes involvement

**Fig. 4:**
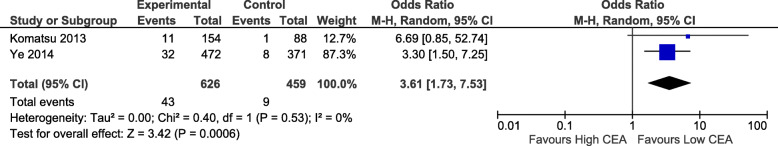
Correlation between CEA level and N2 lymph nodes involvement

All the studies included investigated CEA, while 4 studies also used CYFRA 21-1, and 2 studies used SCC. Given there was a low number of studies looking at tumour markers other than CEA, we excluded the analysis of these markers from our review. The cut-off value for abnormal CEA differed among the studies, ranging from 2.5 to 10 ng/mL (Table 3). Seven out of the twelve articles included in this review had used 5 ng/mL as cut-off value. The majority of the studies involved the use of PET scan (10 studies) for clinical staging, while fewer studies used EBUS (2 studies), mediastinoscopy (1 study), or image guided biopsy (1 study). The most frequently performed procedure was lobectomy (2715, 67.7%), followed by segmentectomy (374, 11.8%), wedge resection (47, 1.5%), pneumonectomy (45, 1.4%),bilobecomy (24, 7.6%). However, 4 studies did not specify the operative procedure. The tumor sub-types were: adenocarcinoma (3622, 77.6%), squamous cell cancer (697, 14.9%), large cell cancer (54, 1.2%), adenosquamous (17, 0.4%), carcinoid (12, 0.3%), other (264, 5.7%) (Table 3). Postoperative staging were specified in 7 studies as the following: stage I (1,228, 79.2%), stage II (121, 7.8%), stage III (198, 12.8%), stage IV (3, 0.2%). Pathologic lymph node status were: NO (2315, 86.3%), N1 (192, 6.7%), N2 (158), N 1–3 (197, 5.5%), while three studies did not report lymph nodes details. Meta-analysis was performed to determine the association of high CEA with death within 5 years and lymph node involvement. High CEA had an odds of death within 5 years that is 3.17 times that of low CEA (95% CI 1.75 to 5.73, *p* = 0.0001). This result had high heterogeneity (chi2 = 67%, *p* = 0.05). This analysis included 3 studies and 1334 patients (Fig. 2). For nodal status, high CEA had a higher odds of there being any positive nodal metastases (OR 3.85, 95% CI 2.64 to 5.62, *p* < 0.00001) compared to low CEA. This result had low heterogeneity (chi2 = 0%, *p* = 0.48). This analysis included 4 studies and 1517 patients (Fig. 3). Further subanalysis revealed that high CEA had higher odds of positive N2 that is 3.61 times that of low CEA (95% CI 1.73 to 7.53, *p* = 0.0006). This analysis included 2 studies and 1085 patients (Fig. 4). Heterogeneity was low (chi2 = 0%, *p* = 0.53).

**Table 3:** Studies included in the review

					Low CEA			High CEA		
NO	Author	Year of publication	No. of patients	CEA cut-off	No. of LN	5 year survival	Recurrence	No. of LN	5 year survival	Recurrence
1	Ryo Maeda [29]	2017	378	5 ng/mL	263 N0, 15 N1–3	87.70%		81 N0, 19 N1–3	75.50%	
2	Yusuke Takahashi [30]	2015	171	5 ng/mL						
3	Gianfranco Buccheri [18]	2003	118	10 ng/mL			16%			70%
4	Riken Kawachi [31]	2009	815	5 ng/mL		76.70%			56.60%	
5	Niels Reinmuth [19]	2002	67	5 ng/mL						
6	Sukki Cho [32]	2013	770	3.5 ng/mL (mean)						
7	Kaoru Kaseda [33]	2016	246	5 ng/mL	168 N0, 19 N1-N2			47 N0, 12 N1-N2		
8	Tatsuya Kato [34]	2013	177	3 ng/mL		93.2% (ADC), 81% (SCC)	6.80%		59.4% (ADC), 51.9% (SCC)	38.80%
9	Terumoto Koike [35]	2012	894	5 ng/mL						
10	Hiroaki Komatsu [36]	2013	279	2.8 ng/mL	87 N0, 1 skip N2			143 N0, 11 skip N2		
11	Yasuhiro Tsutani [37]	2014	100	2.5 ng/mL						
12	Bo Ye [38]	2014	651	5 ng/mL	371 N0, 11 N1, 8 N2			211 N0, 32 N1,18 N2

### Risk of bias assessment

Most of the studies included had good or fair quality score, only 3 studies their quality score was poor. Those whose scored poor on their quality lost some point on follow up.

## Discussion

Accurate preoperative staging of NSCLC is integral for appropriate treatment plan. The main treatment for patients with stage I NSCLC is surgery. Unfortunately, due to limited sensitivity of preoperative imaging, up to 30% of patients with stage I NSCLC may have positive N2-N3 lymph nodes at the time of resection [8, 14, 15]. A meta-analysis of 20 studies showed that mediastinal lymph node staging using CT scan had 57% sensitivity and 82% specificity [16]. Similarly, Cerfolio et al. showed that 7 of 17 patients with cN1 (41%) were found to have positive N2 after lymph node sampling by mediastinoscopy or endoscopic ultrasound with fine needle aspiration (EUS FNA), although N2 involvement was excluded initially by PET/CT scan [17]. The ability to detect subclinical nodal involvement prior to surgery could allow identification of cN0 patients who might benefit from invasive staging, while patients with low CEA levels could conceivably be spared invasive staging if they would otherwise qualify for reasons such as large tumour size or central tumour. Better preoperative staging should result in improved treatment selection, as patients may benefit from neoadjuvant chemotherapy or chemoradiotherapy rather than upfront surgery. In patients with early stage I NSCLC, in whom lymph sampling versus lymph dissection is controversial, the use of CEA could also identify patients in need of more aggressive lymphadenectomy [18–20]. Whether the increased risk of mortality found in this meta-analysis is completely attributable to the increased rate of nodal involvement can not be determined from the available studies’ data, but identification of patients with poor prognosis related to high CEA may also allow for a more tailored approach to post-resection surveillance and patient counselling.

Due to the limited ability of preoperative imaging CT or PET-CT to detect mediastinal lymph nodes disease, interest in serum biomarkers in lung cancer is growing. The most frequently studied tumor marker is carcinoembryonic antigen. All histological types of lung cancer can produce CEA and a role for its use in lung cancer screening and staging was first proposed in the 1970s [21–25]. Recently, studies have demonstrated the usefulness of CEA in patients with NSCLC for postoperative follow up, response to chemotherapy, recurrence, and prognosis. High CEA level has been correlated with advanced disease and poor prognosis. Serum CEA measurement is a simple, non-invasive, inexpensive test. In such case, patients with high CEA level might benefit from lymph node sampling by mediastinoscopy or endoscopic ultrasound with fine needle aspiration (EUS FNA) [18, 25–27]. As shown in our meta-analysis CEA level is correlated with lymph node involvement, and further sub-analysis did reveal that higher CEA associated with positive N2.

There is a discrepancy in the cut-off value of CEA ranges from 2.5 to 10 ng/mL and is attributable to the different techniques used for measurement such as radioimmunoassay (RIA) and enzyme immunoassay [21, 28]. Further studies are needed to standardize the cut-off value. Our meta-analysis about lymph nodes involvement and death within 5 years was limited to few studies because most did not mention the specific details needed to conduct the analysis. Other limitations in our study include the following: the majority of included studies were retrospective, done in a single country (Japan) and many lacked specific lymph nodes details (N0, N1, N2, N3). In addition, we excluded non-English articles. As such we recommend a prospective study using CEA preoperatively to accurately correlate the level of CEA with risk of lymph nodes metastasis, and to determine the cut-off value of CEA.

## Conclusion

There is significant correlation between the CEA level and both nodal involvement and survival. Higher level of CEA is associated with advanced stage, and poor prognosis. Performing preoperative CEA in patient with early stage NSCLC might help to identify patients with more advanced disease which is not detected by imaging, and potentially identify patients for invasive mediastinal lymph node staging, helping to select the most effective therapy for patients with potentially subclinical nodal disease. Further prospective studies are needed to standardize the use of CEA as an adjunct for NSCLC staging.

## Data Availability

Possible upon request, we can share our Excel sheet of data.

## Abbreviations

EBUS: Endobronchial ultrasound
CT: Computed tomography
PET: Positron emission tomography
LN or N: Lymph node
cN: clinical stage lymph node
NSCLC: Non-small cell lung cancer
CEA: Carcinoembryonic antigen
SCC: Squamous cell carcinoma antigen
CYFRA 21-1: Cytokeratin fragment antigen
EUS FNA: Endoscopic ultrasound with fine needle aspiration
RIA: Radioimmunoassay
NOS: Newcastle–Ottawa Scale
